# Detection of normal and slow saccades using implicit piecewise polynomial approximation

**DOI:** 10.1167/jov.21.6.8

**Published:** 2021-06-14

**Authors:** Weiwei Dai, Ivan Selesnick, John-Ross Rizzo, Janet Rucker, Todd Hudson

**Affiliations:** 1 Department of Electrical and Computer Engineering, Tandon School of Engineering, New York University, Brooklyn, NY, USA; 2 Department of Neurology, School of Medicine, New York University, New York, NY, USA

**Keywords:** saccade detection, slow saccades, saccade quantitative analysis

## Abstract

The quantitative analysis of saccades in eye movement data unveils information associated with intention, cognition, and health status. Abnormally slow saccades are indicative of neurological disorders and often imply a specific pathological disturbance. However, conventional saccade detection algorithms are not designed to detect slow saccades, and are correspondingly unreliable when saccades are unusually slow. In this article, we propose an algorithm that is effective for the detection of both normal and slow saccades. The proposed algorithm is partly based on modeling saccadic waveforms as piecewise-quadratic signals. The algorithm first decreases noise in acquired eye-tracking data using optimization to minimize a prescribed objective function, then uses velocity thresholding to detect saccades. Using both simulated saccades and real saccades generated by healthy subjects and patients, we evaluate the performance of the proposed algorithm and 10 other detection algorithms. We show the proposed algorithm is more accurate in detecting both normal and slow saccades than other algorithms.

## Introduction

An effective and reliable saccade detection algorithm is essential for eye movement studies. Saccadic eye movements are fast brief eye movements that rapidly redirect our line of sight to visual targets (Leigh & Zee, 2015). Advances in the study of brain anatomy and the pathology and of eye movements have reinforced the substantial utility of saccades and saccade detection in research (Ramat et al., 2007). Video-based eye-tracking systems aid the study of eye movements, due to their ease of use, accessibility, noninvasiveness, and relative low cost. The analyses of saccades have been applied to study autism (Shic et al., 2008), cognition (Federici & Mele, 2012), concussion (Rizzo et al., 2016b), multiple sclerosis (Hainline et al., 2017), and many other behavioral, cognitive, and neurological problems. Accurate identification of saccades in time-series data produced by eye-tracking systems is a critical step in eye movement studies. Although manual labelling of saccades in data by experts has been considered reliable, it is tedious and can take days to label data that took only minutes to record (Monty, 1975). Furthermore, classifications between experts may differ significantly (Hooge et al., 2018). An effective and reliable saccade detection algorithm is needed.

The accurate detection of saccadic abnormalities offers important clues in the diagnosis of numerous disorders and may provide opportunities for timely diagnosis and treatment (Ramat et al., 2008; Termsarasab et al., 2015). Slow saccades are an indication of neurological disorders and often imply a specific pathological disturbance (Marx et al., 2012). Normal saccades follow a known relationship between saccade peak velocity and amplitude. In this relationship, called a “main sequence” (Bahill et al., 1975), the peak saccadic velocity increases linearly as a function of saccadic amplitude for small-amplitude saccades, then gradually saturates at larger amplitudes. This “main sequence” curve varies among, individuals but is highly reproducible for an individual (Gangemi et al., 1991). Main sequence curves that fall outside the “normal range” are critical to neurological diagnosis and often may be the most specific examination finding guiding diagnostic evaluation. Abnormally slow saccades, in the absence of a definite extraocular muscle or cranial nerve disorder, are suggestive of diseases involving brainstem saccadic burst neurons (Baloh et al., 1975; Barton et al., 2003; Horn & Büttner-Ennever, 1998; Kaneko, 1996). Lesions in the frontal eye field (Dias & Segraves, 1999) and dorsolateral prefrontal cortex (Koval et al., 2013) can demonstrate a slowing in peak saccadic velocity; however, this is not commonly seen clinically. Examples of conditions that typically cause saccadic slowing include progressive supranuclear palsy (PSP) (Chen et al., 2010; Garbutt et al., 2003), spinocerebellar ataxia type 2 (Wadia & Swami, 1971), and Huntington's disease (Lasker et al., 1988). Note that not only do such disorders cause slow saccades, but in some instances, such as PSP, the diagnosis depends on the identification of slow saccades. Additional factors, such as saccade adaptation and visual salience, may also affect saccade peak velocities (Schütz et al., 2011); even subtle decreases in the peak velocity of otherwise normal saccades have been observed in states of mental fatigue (Di Stasi et al., 2012). Thus, the accurate detection of slow saccades is a critical component in our understanding of normal and pathological saccadic behavior.

**Figure 1.**
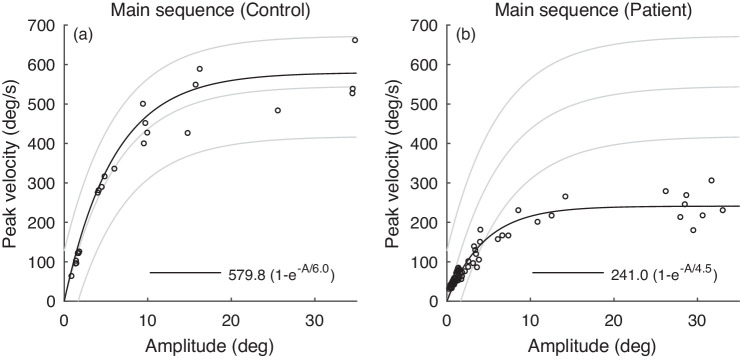
Saccade main sequence (peak velocity vs. saccade amplitude) for two individuals. (a) An individual with normal saccades. (b) An individual with slow saccades. The gray lines show the normative range for the main sequence as observed in the laboratory of the authors.

Figure 1 shows the saccade main sequence data (peak velocity versus amplitude) of two individuals, one of whom exhibits slow saccades. The exponential formula

(1) $$V_{p}=\eta \left(1-e^{-A/c}\right),$$

was proposed by Baloh to model main sequence data (Baloh et al., 1975). This formula models the relationship between the peak velocity (designated $V_{p}$) and the saccade amplitude (designated A). In the (formula 1), the parameter η represents the maximum attainable peak angular velocity of any saccade made by the individual, and the parameter c determines the proportionality constant between $V_{p}$ and A for small saccades. We find the parameters of the exponential curve for each individual in Figure 1 using the function fitnlm in MATLAB for nonlinear regression.

Existing saccade detection algorithms work well for the detection of normal saccades (for which they are mainly developed). However, they often fail to detect slow saccades. This is because the lower velocity of slow saccades makes velocity thresholding (VT) less reliable. The influence of noise is more problematic for the detection of slow saccades than for normal saccades of the same amplitude, especially since temporal differentiation (to determine velocity from position) amplifies noise. Thus, improved noise reduction (denoising) is needed. The proposed method comprises mainly a new denoising step.

We propose an algorithm to detect both normal and slow saccades with high accuracy and robustness. The algorithm consists of two steps: (1) nonlinear smoothing of the time-series recording generated by an eye-tracking device, and (2) simple VT to detect saccades. The algorithm is based on an implicit piecewise polynomial model for a time-series containing saccadic eye movements. Conceptually, the waveform of a saccade can be approximately modeled as a piecewise quadratic signal, so its third-order temporal derivative is sparse (exhibits few non-zero values). At the same time, the first-order temporal derivative is also sparse because the velocity is zero when the eye is not moving. Therefore, we prescribe a generalization of total variation (TV) regularization (Rudin et al., 1992) to capture these properties, leading to the processed time-series data exhibiting approximately constant-valued segments interspersed with approximately piecewise-quadratic segments. The algorithm then uses simple VT to detect saccadic eye movements in the nonlinearly processed time-series.

In this article, we demonstrate the proposed algorithm is more accurate in detecting both normal and slow saccades than other algorithms. We use both simulated saccades and real saccades produced by healthy subjects and patients to evaluate the performance of saccade detection using the proposed algorithm and 10 other saccade detection algorithms. Simulated saccades are generated at various sampling rates using a parametric model for saccadic eye movements (Dai et al., 2016). Eye-tracking time-series from healthy subjects are publicly available and saccades were manually labelled by two eye movement experts (Larsson et al., 2013; Nyström & Holmqvist, 2010). The eye movements of five patients with neurological diseases causing saccade slowing were recorded in our laboratory (Rizzo et al., 2016a). A MATLAB implementation of the proposed algorithm is made available online by the authors. All algorithmic parameters are determined within the method (i.e., without input from the user).

## Current algorithms for saccade detection

Many saccade detection algorithms have been developed to facilitate the quantitative analysis of eye movements. We review some popular algorithms designed to distinguish saccades and fixations. This review is intended to cover the major classes of saccade detection algorithms; not every algorithm, because they are too numerous given the growing interest in eye movements (Titz et al., 2018). We do not consider smooth pursuit eye movements, which must be initiated by a moving target (Leigh & Zee, 2015), and are therefore absent in eye-tracking time-series when the subject is reading text, scanning an image, or engaging in functional saccade tests. Moreover, we consider the detection of saccades in data from eye trackers that are world fixed, rather than wearable eye trackers, a distinction which should be noted (Hessels et al., 2018).

Dispersion thresholding (DT) algorithms classify points in an eye-tracking time-series as a fixation when the eye lingers in a small area, and as a saccade otherwise. Algorithms of this type are integrated into many commercial eye-tracking software systems. Various methods for calculating dispersion and setting parameters have been proposed (Blignaut, 2009). However, this type of method is sensitive to noise and drift in the data; and they do not accurately estimate the start-time and the end-time of saccades.

VT algorithms are the most commonly used for saccade detection (Komogortsev et al., 2010; Salvucci & Goldberg, 2000). This type of algorithm classifies points as a saccade if their velocity is greater than a threshold, and as a fixation otherwise. Since saccadic eye movements are the fastest eye movement, it is natural to apply VT to distinguish saccades and fixations. In the basic approach, one manually sets a fixed velocity threshold for saccade detection (Bahill et al., 1981). However, a low threshold value leads to many false detections owing to noise, whereas a high threshold value leads to many saccades being missed. Thus, instead of a fixed velocity threshold value, methods with adaptive thresholding have been proposed (Mould et al., 2012). Engbert set the value of the velocity threshold to be proportional to the standard deviation of the velocity data (Engbert & Kliegl, 2003; Engbert & Mergenthaler, 2006). We consider this as an adaptive global velocity threshold. Although the method was originally proposed for microsaccade detection, it was found to be useful for the detection of larger saccades as well. Extending Engbert's work, Nystrom developed an algorithm which finds an adaptive diaeresis global velocity threshold for saccade occurrence and adaptive local velocity thresholds for the end of each saccade (Holmqvist & Nyström, 2010). Furthermore, a modified version of Nystrom's algorithm was recently developed by (Friedman et al., 2018).

Acceleration-based–algorithms have also been proposed for saccade detection. This type of algorithm is based on the observation that the velocity at the start and end of a saccade changes much faster than it does for other types of eye movements (Behrens & Weiss, 1992; Behrens et al., 2010). However, this type of algorithm tends to be highly sensitive to noise, because it requires two instances of numerical temporal differentiation of the time-series, and each instance increases the noise level. The analysis software of EyeLink (SR Research Ltd, Kanata, Ontario, Canada) applies velocity and acceleration thresholding together to detect saccades.

Machine-learning–based algorithms have been proposed more recently, which detect saccades based on features extracted from the data. König calculates distance, velocity, acceleration, and angular velocity of each sample, and uses k-means clustering to distinguish saccades and fixations (Buffalo & König, 2014). Otero-Millan applies k-means clustering to facilitate the detection of microsaccades (Otero-Millan et al., 2014). Zemblys uses a Rayleigh test (Larsson et al., 2015), i2mc (Hessels et al., 2017) and eight other features extracted from the data, and trains a random forest model to classify saccades and other eye movements (Zemblys et al., 2018). Machine-learning based algorithms have shown superior performance of saccade detection compared to hand-crafted algorithms (Zemblys et al., 2018). However, machine-learning models can suffer from overfitting problems and need labelled data for training.

Other types of saccade detection algorithms have also been proposed, but are not as commonly used as dispersion or velocity-based methods (Daye & Optican, 2014; Santini et al., 2016). A hidden Markov model (HMM) method uses a finite state machine with two states (one for the velocity distribution of saccades, one for fixations) that attempts to determine the most likely classification of each point (Komogortsev et al., 2010). A minimum spanning tree (MST) method constructs an MST and uses Prime's algorithm to find edges whose length are longer than a prescribed value to be identified as saccades, and identifies fixations as clusters of points separated by saccades. A Kalman filter (KF) method uses a two-state KF to classify eye movements (Sauter et al., 1991). The idea behind this algorithm is that nonsaccadic eye movements can be modeled fairly accurately by a simple model, and that saccadic movements follow a sufficiently different model so they can be distinguished by a hypothesis test. A linear regression-based method segments a time-series into blocks and classifies each block using a HMM (Pekkanen & Lappi, 2017).

## Parametric saccade model

We previously proposed a parametric model for saccadic eye movement, which can be used to simulate saccades and for the evaluation of saccade detection algorithms (Dai et al., 2016). The model has three parameters: η, c, and A. The formula for the saccade model is

(2) s(t)=cf(ηt/c)-cf(ηt/c-A/c)

where f is the function defined as

(3) $$f\left(t\right)=\left\{\begin{array}{cc} t+0.25e^{-2t}, & t\geq 0\\ 0.25e^{2t}, & t\leq 0. \end{array}\right.$$

The function f is a “soft ramp” function. The parameters η and c control the shape of the “main sequence” (formula 1). In the (formula 2), the parameters η and c serve to scale the function f.

We use this model to simulate an eye-movement time-series comprising saccades and fixations so that we can quantitatively compare the performance of various saccade detection algorithms. Although the simulated data do not simulate all aspects of real eye-tracking data (e.g., there are no post-saccadic oscillations), by adding noise to the simulated data, we can measure the sensitivity of the methods to noise. Noise is a significant issue because VT requires temporal differentiation of the position data to determine the velocity, but temporal differentiation amplifies noise which hinders reliable detection.

We also use eye-tracking time-series with saccades labeled by eye movement experts. But, as recognized, even experts can differ in their labeling of a given noisy eye-movement time-series (Andersson et al., 2017; Hooge et al., 2018). Evaluations of the performance and accuracy of algorithms, based on expert annotations, should be interpreted with caution.

## Proposed algorithm for saccade detection

The proposed algorithm consists of two steps: (1) nonlinear filtering of eye-movement time-series to reduce noise, and (2) VT to detect saccades.

We propose a nonlinear filtering method based on sparse time-series properties. Namely, in the absence of noise, the first-order and higher-order temporal derivatives of eye-movement time-series can be modeled as sparse (i.e., consisting mostly of zero values). We model the recorded time-series $y=(y_{1},y_{2},\cdots ,y_{N})$ as

(4) y=x+w

where $x=(x_{1},x_{2},\cdots ,x_{N})$ is a time-series comprising saccades and fixations, and $w=(w_{1},w_{2},\cdots ,w_{N})$ is additive white Gaussian noise. It has been shown that eye trackers generally produce white noise (Coey et al., 2012; Wang et al., 2016).

**Figure 2.**
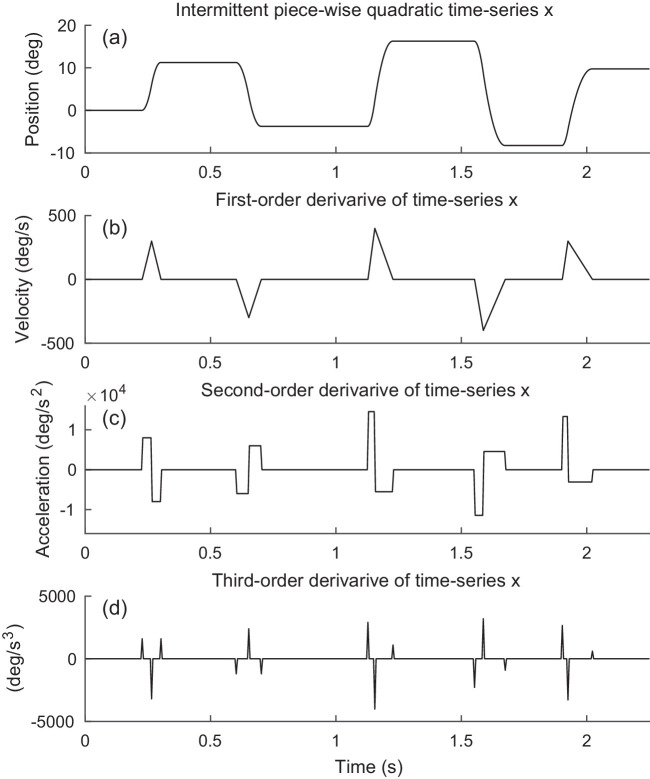
Example of an intermittent piecewise quadratic signal x and its derivatives. (a) The signal comprises constant-valued segments separated by piecewise quadratic segments. (b) The first-order derivative comprises zero-valued segments separated by piecewise linear segments. (c) The second-order derivative is piecewise constant. (d) The third-order derivative is sparse, only a few values are non-zero.

We make two observations and assumptions about eye-movement time-series: 1) Saccadic eye movements are rapid and have short duration (<80 milliseconds). The eye remains fairly stable during fixations. Therefore, the first-order derivative of the time-series is sparse, that is, the angular velocity of the eye is mostly zero. 2) The waveform of a saccadic eye movement can be modeled as approximately piecewise quadratic; hence, its third-order temporal derivative is sparse. Figure 2 shows an example of a time-series that is mostly constant, with intervals where it is piecewise quadratic. We name this an intermittent piecewise quadratic signal. The example time-series shown in Figure 2 shows a simulated eye-movement time-series comprising five saccades and six fixations.

From a noisy eye-movement time-series y, we estimate the time-series x by minimizing the objective function

(5) $$J\left(x\right)=\frac{1}{2}{∥y-x∥}_{2}^{2}+\alpha ∥D_{1}{x∥}_{1}+\beta {∥D_{3}x∥}_{1}$$

where $D_{1}$ is the first-order difference operator and $D_{3}$ is the third-order difference operator,

(6) $$\begin{array}{c} \begin{array}{c} D_{1}=\left[\begin{array}{ccccc} -1 & 1 & & & \\ & -1 & 1 & & \\ & & \ddots & \ddots & \\ & & & -1 & 1 \end{array}\right],\\ D_{3}=\left[\begin{array}{ccccccc} -1 & 3 & -3 & 1 & & & \\ & -1 & 3 & -3 & 1 & & \\ & & \ddots & \ddots & \ddots & \ddots & \\ & & & -1 & 3 & -3 & 1 \end{array}\right] \end{array}\; \end{array}$$

The quadratic term and the L1 norm terms are defined via

(7) $$\begin{array}{c} {∥x∥}_{2}^{2}=x_{1}^{2}+x_{2}^{2}+\cdots +x_{n}^{2},\\ {∥x∥}_{1}=|x_{1}\left|+\right|x_{2}\left|+\cdots +\right|x_{n}| \end{array}$$

respectively, and α and β are regularization parameters.

The first term ${∥y-x∥}_{2}^{2}$ in (5) causes the estimated time-series x to be similar to the noisy time-series y. The second and third terms, $∥D_{1}{x∥}_{1}$ and $∥D_{3}{x∥}_{1}$, induce sparsity of the first-order and third-order derivatives of the estimated time-series x. The quadratic error corresponds with the assumption that the observed time-series y is an observation of the unknown time-series x that has been corrupted by additive zero-mean white Gaussian noise; while the L1 norm terms are to induce sparsity (Chen et al., 1998; Hyvärinen et al., 2001).

This formulation assumes the underlying time-series x comprises mostly constant-valued segments, with some segments that are piecewise quadratic (Figure 2).

The minimization of the objective (function 5) reduces to TV denoising (Rudin et al., 1992) when β=0. Although TV denoising is appropriate for the denoising of piecewise constant time-series, it leads to artifacts when used for the denoising of other types of time-series. Generalizations of TV using high-order difference operators have been proposed for more general time-series (Bredies et al., 2010; Chan et al., 2000; Sanders et al., 2017; Stefan et al., 2010). For example, the effectiveness of sparse higher-order derivatives has been demonstrated for the processing of chromatograms (Ning et al., 2014). The objective (function 5) is a particular generalization of TV denoising that we propose for the denoising of eye-movement time-series.

**Figure 3.**
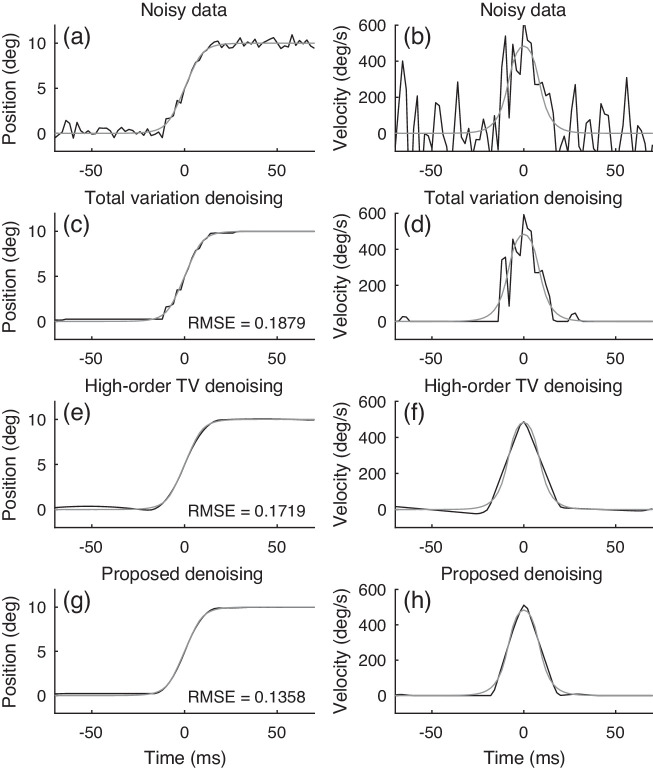
Denoising a simulated saccadic eye movement using total variation (sparse first-order derivative), high-order TV (sparse third-order derivative), and the proposed algorithm (sparse first-order and third order-derivatives). The root mean square error (RMSE) value is indicated in each graph. The calculated velocity time-series are shown on the right. In each graph, the gray line depicts the noise-free (ideal) time-series. Black lines depict the noisy signal (a, b) and denoised signals (c–h).

Figure 3 illustrates the superior performance of the proposed form of denoising compared with other types of TV denoising. We use the parametric saccade (model 2) to simulate a saccadic time-series. We add white Gaussian noise (σ=0.4) to simulate a noisy time-series (Figure 3(a)). We conduct denoising using TV, high-order TV, and by minimizing the proposed objective (function 5). We set the parameters of each algorithm to minimize the root mean square error (RMSE) between the denoised and noise-free time-series, so that each algorithm performs at its best. TV denoising exhibits stair-case artifacts (Figure 3(c)). High-order TV denoising produces a smooth result but does not preserve the constant-valued intersaccadic behavior of the time-series (Figure 3(e)). The proposed algorithm achieves the lowest (best) RMSE value. It also yields a velocity value of zero away from a saccade, which is important for the subsequent detection of saccades by simple VT.

To develop an algorithm to minimize the objective (function 5), we use the iterative majorization-minimization approach. This approach minimizes a sequence of functions $G^{\left(k\right)}\left(x\right)$ each of which is a majorizer of J(x). The majorizer functions $G^{\left(k\right)}\left(x\right)$ are selected so as to be easier to minimize than objective function J(x). Each function $G^{\left(k\right)}\left(x\right)$ should be chosen to satisfy

(8) $$\begin{array}{c} G^{\left(k\right)}\left(x\right)\geq J\left(x\right),\;\forall x\; \end{array}$$

(9) $$\begin{array}{c} G^{\left(k\right)}\left(x^{\left(k\right)}\right)=J\left(x^{\left(k\right)}\right)\; \end{array}$$

where $x^{\left(k\right)}$ denotes x at iteration k.

For the majorizer of the L1 norm, we use the differentiable quadratic function

(10) $$\;\;\;\;\;\;\;\;\;\;\;\;\;\;\frac{1}{2}x^{T}\Lambda^{\left(k\right)}x\;+\;\frac{1}{2}∥x^{\left(k\right)}{∥}_{1}{\;\geq \;∥x∥}_{1},\;\Lambda^{\left(k\right)}\;=\;\mathrm{diag}(1/|x^{\left(k\right)}\left|\right)\;\;\;$$

where $x^{T}$ designates the transpose of the vector x. Also, by diag(1/v), where v is a vector, we mean the diagonal matrix whose diagonal elements are the reciprocals of the elements of the vector v. To avoid numerical problems when elements of $x^{\left(k\right)}$ are equal to zero, we actually use $\Lambda^{\left(k\right)}=\mathrm{diag}(1/(|x^{\left(k\right)}\left|+\varepsilon )\right)$ with $\varepsilon =10^{-10}$ (Ding & Selesnick, 2016).

The majorizer of the objective (function 5) is then given by

(11) $$\begin{array}{ccc} G^{\left(k\right)}\left(x\right) & \;= & \frac{1}{2}{∥y-x∥}_{2}^{2}+\frac{1}{2}\alpha x^{T}D_{1}^{T}\Lambda_{1}^{\left(k\right)}D_{1}x\\ & & +\;\frac{1}{2}\beta x^{T}D_{3}^{T}\Lambda_{3}^{\left(k\right)}D_{3}x+C \end{array}$$

where

(12) $$\begin{array}{c} \Lambda_{1}^{\left(k\right)}:=\mathrm{diag}(1/(|D_{1}x^{\left(k\right)}|+\varepsilon ))\; \end{array}$$

(13) $$\begin{array}{c} \Lambda_{3}^{\left(k\right)}:=\mathrm{diag}(1/(|D_{3}x^{\left(k\right)}|+\varepsilon ))\; \end{array}$$

and where C does not depend on x. The minimizer $x^{\left(k+1\right)}$

(14) $$x^{\left(k+1\right)}:=\arg\min_{x}G^{\left(k\right)}\left(x\right)$$

is given by the matrix equation

(15) $$\;\;\;\;\;\;\;\;\;x^{\left(k+1\right)}={\left(I+\alpha D_{1}^{T}\Lambda_{1}^{\left(k\right)}D_{1}+\beta D_{3}^{T}\Lambda_{3}^{\left(k\right)}D_{3}\right)}^{-1}y$$

for k=0,1,2,⋯,K-1 where K is the number of iterations, and I is the identity matrix. The sequence of minimizers $x^{\left(k\right)}$ converges to the minimizer of (5) because the objective function is convex (Figueiredo et al., 2007). The resulting time-series, x will be approximately intermittent piecewise quadratic, and constitutes the output of a nonlinear filter with y as the input time-series.

**Figure 4.**
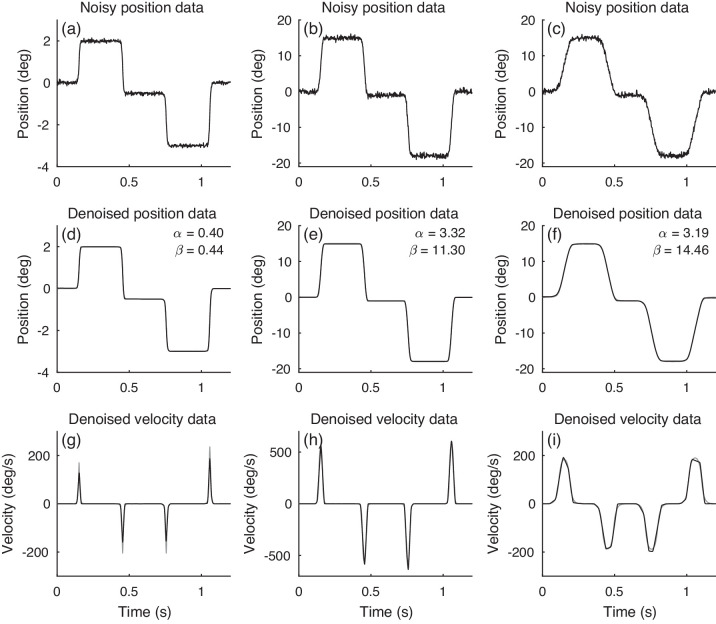
Parameters α and β are set according to the estimated noise level, average saccade amplitude, and average saccade duration in the data. The proposed algorithm can recover data well with automatic parameter setting. Black lines depict the noisy signals (a, b, c) and denoised signals (d–i). Gray lines are noise-free signals (d–i).

The second step of the algorithm is to apply VT to the velocity (first-order temporal derivative) of the estimated time-series x to detect saccadic eye movements. The velocity is calculated using a central difference filter; the coefficients are [0.5,0,-0.5]. The velocity is zero during fixations and piecewise linear during saccades (Figure 4). We count a saccade as being detected when the velocity exceeds 30 degrees/second.

## Parameter setting

The proposed method requires that the parameters α and β be prescribed. We note that increasing the parameter α increases the sparsity of the estimated velocity time-series, whereas the value of parameter β increases the sparsity of the derivative of the acceleration (which also affects the shape of the estimated velocity profiles). We provide a software-based interactive graphical user interface (GUI) to illustrate the influence of α and β with various noise levels and saccade shapes. (See https://youtu.be/mwqk0uQc3is.)

To find empirical formulas to set parameters α and β, we simulated saccades (at a sampling rate of 500 samples/second) of various amplitudes using the parametric saccade model and we considered the addition of white Gaussian noise of various standard deviations σ. For each saccade amplitude and σ value, we created 100 noise realizations. Using a fine grid for α and β, we found the values of α and β giving the least MSE for the noise reduction algorithm. Although the values so-obtained do not follow a simple formula exactly, based on these results, we suggest setting

(16) α=8σ

provided β can also be suitably set. We find that this choice for setting α is effective even when β varies over a small range.

To consider the question of how to set the parameter β, the trade-off is as follows. A too-low value of the parameter β leads to insufficient noise reduction and a high rate of false detections due to the estimated velocity time-series being too noisy. In contrast, a too-high value of β leads to an over-smoothing of the saccade waveform, which means a low and wide estimate of saccade velocity profiles; this can cause saccades not to be detected or their duration to be over estimated. Therefore, for a time-series with many small saccades, we should use a smaller value of β so as to reliably detect them. For a time-series with large slow saccades, we can use a greater value of β. Thus, we choose to set β to increase with increasing average saccade amplitude and duration. After empirical study, we suggest setting

(17) $$\beta =4\sqrt{A}e^{5D}\sigma$$

where σ is the estimated noise, and A and D are estimates of the average saccade amplitude and duration in the time-series. The relation of parameter β to the square root of A and the exponential of D was found through experimentation, and found to be reliable when processing eye-tracking data consist of many different saccades. Note that, for normal eye-movement data, the parameters α and β are within predictable bounds because the amplitudes and durations of normal saccades themselves are within predictable bounds (i.e., amplitudes are usually less than 30 degrees and durations usually less than 100 milliseconds).

The estimate of σ, A, and D do not need to be precise because small changes in these parameters do not change the results significantly. We estimate them as follows: We first apply a low-pass differentiator with a cut-off frequency of 10 Hz to obtain the velocity of data. We then classify candidate saccades as those for which the velocity exceeds 10 degrees/second and for which the duration is at least 12 milliseconds. We combine two saccades into one when the interval between them is less than 20 samples in this noise estimation process. We estimate the noise level σ as the standard deviation of the centered fixation data. We set the average saccade amplitude A to be the sum of saccade amplitudes divided by the number of saccades; we set the average saccade duration D similarly.

We demonstrate the performance of the proposed parameter setting in Figure 4. The time-series consist of four simulated small normal saccades (Figure 4(a)), four large normal saccades (Figure 4(b)), and four large pathological slow saccades (Figure 4(c)). White Gaussian noise is added to the simulated time-series. The result in Figure 4 shows that method sets the parameters according to the input data and provides reliable denoising. No parameter needs to be tuned by users.

The parameters α and β should also be adjusted according to the time-series sampling rate, f. We simulate saccadic time-series at 250 samples/second, 500 samples/second, and 1000 samples/second using the parametric saccade model. Based on experimental results, we set

(18) $$\alpha =0.016f\sigma ,\;\beta =0.008f\sqrt{A}e^{5D}\sigma$$

for f≤500 samples/second, and

(19) $$\begin{array}{c} \;\;\;\;\;\;\;\;\;\;\;\;\;\alpha =\left(0.0032f+6.4\right)\sigma ,\;\beta =\left(0.0016f+3.2\right)\sqrt{A}e^{5D}\sigma \;\;\;\; \end{array}$$

for f>500 samples/second.

We define saccade onset as the point when the velocity exceeds 30 degrees/second. We define the end of a saccade as the point when the velocity falls below 10 degrees/second. We use a lower velocity threshold value for the end of a saccade because the saccade velocity profiles (especially large saccades) are usually asymmetric, with higher acceleration and lower deceleration. We use fixed VT for the proposed method because it is simpler than adaptive VT. Adaptive thresholding has negligible benefit here because of the high noise suppression of the proposed method. (Adaptive thresholding is most useful when a signal is somewhat noisy.)

We use several postprocessing rules to trim out some false positives (e.g., candidate saccades that are not plausible), as is done in other existing methods. Any detected saccade is discarded if it is fewer than 10 samples away from a blink. The minimal duration of a saccade is set to be 12 milliseconds. This is a conservative constraint to avoid irregular artifacts in the recorded time-series. However, it can be changed for the study of microsaccades. We use a minimum inter-saccadic interval of 40 milliseconds so as to ignore post-saccadic oscillations; they are not our focus. Finally, any detected saccade with a peak velocity of more than 800 degrees/second is discarded because such high speeds are not physiologically plausible.

Compared with the other algorithms to be considered, the proposed method generates fewer false positives. Therefore, these post-processing steps do not make a significant difference for the proposed method, and the assumptions above (e.g., a saccade must be of at least 12 milliseconds in duration) are not critical for the proposed method. We use the same post-processing steps for other existing saccade detection algorithms for the purpose of comparing the performance of multiple algorithms. Note that both the proposed and existing algorithms will still generally have some false positives that are not removed by these post-processing steps.

## Experimental evaluation

We compare the proposed algorithm with ten other saccade detection algorithms using simulated data, eye-tracking data provided by (Holmqvist & Nyström, 2010; Larsson et al., 2013), and eye-tracking data of pathological slow eye movements recored in our laboratory (Rizzo et al., 2016a). The methods being compared include velocity-based algorithms proposed by Engbert and Kliegl (2003), Holmqvist and Nyström (2010), Friedman et al. (2018), and VT; machine learning-based algorithms proposed by Buffalo and König (2014), and Zemblys et al. (2018); DT, HMM, MST, and KF. For algorithms VT and DT, we use our own software implementation. For the other algorithms, we use the software provided by the authors (Engbert & Kliegl, 2003; Friedman et al., 2018; Holmqvist & Nyström, 2010; Komogortsev et al., 2010; König & Buffalo, 2014; Zemblys et al., 2018). We use F1 score, precision, recall, true-positive rate, false-positives, and false-negative rate to measure the accuracy of all saccade detection algorithms. They are defined as

(20) $$\begin{array}{c} \;\;\;\;\;\;\;\;\;\;\;\;\;\text{F1}\;\text{score}=2\;\frac{\text{Precision}\cdot \text{Recall}}{\text{Precision}+\text{Recall}}\; \end{array}$$

(21) $$\begin{array}{c} \;\;\;\;\;\;\;\;\;\;\;\;\;\text{Precision}=\frac{\text{True}\;\text{positive}}{\text{True}\;\text{positive}+\text{False}\;\text{positive}}\; \end{array}$$

(22) $$\begin{array}{c} \;\;\;\;\;\;\;\;\;\;\;\;\;\text{Recall}=\frac{\text{True}\;\text{positive}}{\text{True}\;\text{positive}+\text{False}\;\text{negative}}\; \end{array}$$

(23) $$\begin{array}{c} \;\;\;\;\;\;\;\;\;\;\;\;\;\text{True}\;\text{positive}\;\text{rate}=\frac{\text{True}\;\text{positive}}{\text{Number}\;\text{of}\;\text{saccades}\;\text{in}\;\text{data}}\; \end{array}$$

(24) $$\begin{array}{c} \;\;\;\;\;\;\;\;\;\;\;\;\;\text{False}\;\text{negative}\;\text{rate}=\frac{\text{False}\;\text{negative}}{\text{Number}\;\text{of}\;\text{saccades}\;\text{in}\;\text{data}}.\;\;\; \end{array}$$

We implement an event-by-event comparison using the software provided by Warby et al. (2014). A true positive is defined if there is an overlap in time between a labeled saccade and a detected saccade. This is a more reliable comparison because the precise onset and offset (end of saccades) are difficult to manually indicate with high precision using a mouse-driven graphical user interface.

We first show, using simulated time-series, that the proposed algorithm detects saccades more accurately even when the time-series is very noisy. We simulate eye-movement time-series (at 500 samples/second) consisting of fixations and 50 saccades of various amplitudes. We add white Gaussian noise with various standard deviation σ to simulate different noise levels.

**Figure 5.**
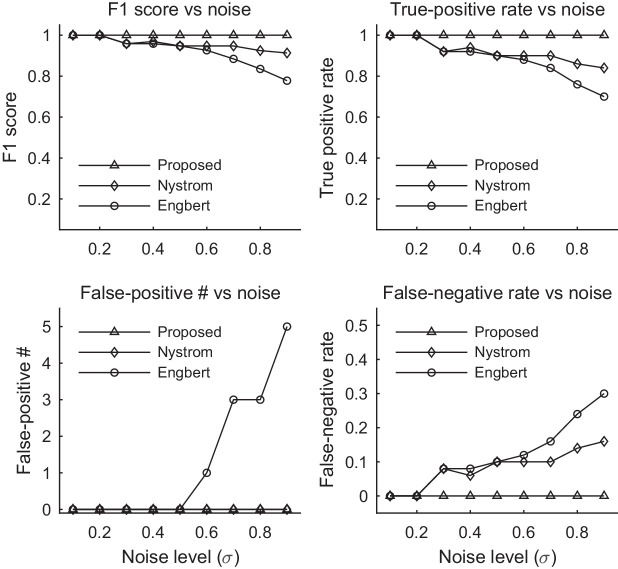
Evaluation of saccade detection algorithms proposed here and by Nystrom and Engbert. The saccades are simulated. Algorithms by Nystrom and Engbert miss more true saccades and detect more false saccades at higher noise levels.

Table 1 shows that the algorithms by Nystrom, Engbert, Zemblys, Friedman, and König can detect saccades well when the noise in the time-series is low. However, these algorithms tend to perform worse at higher noise levels. As shown in Figure 5, velocity-based algorithms (Nystrom's and Engbert's) miss more true saccades and detect many false saccades at high noise levels. The proposed and Zembly's algorithm are shown to be capable of detecting saccades correctly when the data are quite noisy.

**Table 1.** Evaluation of saccade detection algorithms using simulated time-series (500 samples/second). F1 scores show the proposed algorithms detects the saccades perfectly under various noise levels. Zemblys’ algorithm also detect saccades correctly under various noise levels. Other algorithms tend to perform worse at higher noise levels.

	Proposed	Zemblys	Nystrom	König	Engbert	VT	DT	HMM	Friedman	MST	KF
σ=0.1	1.00	1.00	1.00	0.96	1.00	0.18	0.78	0.23	0.98	0.63	0.49
σ=0.2	1.00	1.00	1.00	0.96	1.00	0.40	0.51	0.18	0.85	0.50	0.04
σ=0.3	1.00	1.00	0.96	0.96	0.96	0.47	0.27	0.28	0.45	0.49	0.04
σ=0.4	1.00	0.98	0.97	0.96	0.96	0.58	0.27	0.04	0.21	0.22	0.04
σ=0.5	1.00	1.00	0.95	0.96	0.95	0.61	0.26	0.04	0.07	0.15	0.04
σ=0.6	1.00	1.00	0.95	0.95	0.93	0.59	0.27	0.04	0.02	0.11	0.04
σ=0.7	1.00	1.00	0.95	0.94	0.88	0.62	0.26	0.76	0.03	0.07	0.04
σ=0.8	1.00	1.00	0.92	0.93	0.84	0.55	0.27	0.76	0.00	0.06	0.04
σ=0.9	1.00	0.98	0.91	0.95	0.78	0.54	0.25	0.76	0.00	0.04	0.04
mean	1.000	0.996	0.956	0.950	0.921	0.504	0.348	0.342	0.289	0.252	0.089

We also evaluate saccade detection algorithms when the time-series are at various sampling rates. The same eye movement time-series as above were simulated, but with the sampling rates of 250 and 1,000 samples/second. The results are listed in Table 2 and Table 3. The proposed algorithm accurately detects saccades in time-series at various sampling rates. The proposed and Zemblys’ algorithms outperform other existing saccade detection algorithms.

**Table 2.** Evaluation of saccade detection algorithms using simulated time-series (250 samples/second). F1 scores show the proposed algorithm outperforms other existing algorithms. Zemblys’ algorithm also detect saccades correctly under various noise levels. Other algorithms tend to perform worse at higher noise levels.

	Proposed	Zemblys	König	Nystrom	Engbert	Friedman	VT	MST	DT	HMM	KF
σ=0.1	1.00	1.00	0.96	1.00	1.00	0.89	0.19	0.82	0.84	0.16	0.27
σ=0.2	1.00	1.00	0.96	0.98	0.96	0.78	0.24	0.64	0.28	0.41	0.52
σ=0.3	1.00	1.00	0.96	0.96	0.95	0.76	0.35	0.50	0.28	0.39	0.08
σ=0.4	1.00	1.00	0.95	0.94	0.88	0.66	0.45	0.39	0.25	0.26	0.04
σ=0.5	1.00	1.00	0.95	0.92	0.82	0.51	0.45	0.22	0.28	0.11	0.04
σ=0.6	0.98	1.00	0.95	0.88	0.78	0.40	0.55	0.18	0.26	0.11	0.04
σ=0.7	0.99	0.98	0.89	0.82	0.60	0.29	0.57	0.13	0.24	0.08	0.04
σ=0.8	0.98	0.97	0.87	0.80	0.53	0.29	0.54	0.10	0.27	0.04	0.04
σ=0.9	0.99	0.98	0.83	0.57	0.40	0.29	0.48	0.08	0.27	0.04	0.04
mean	0.993	0.992	0.923	0.874	0.768	0.540	0.423	0.342	0.329	0.178	0.122

**Table 3.** Evaluation of saccade detection algorithms using simulated time-series (1000 samples/second). F1 scores show the proposed algorithm detects saccades perfectly under various noise levels. Zemblys’ algorithm also detects saccades correctly under various noise levels. Other algorithms tend to perform worse at higher noise levels.

	Proposed	Zemblys	Nystrom	König	Engbert	HMM	DT	Friedman	VT	KF	MST
σ=0.1	1.00	1.00	1.00	0.96	1.00	0.36	0.77	0.99	0.31	0.08	0.00
σ=0.2	1.00	1.00	1.00	0.96	1.00	0.11	0.81	0.88	0.46	0.04	0.00
σ=0.3	1.00	1.00	1.00	0.96	0.98	0.11	0.51	0.00	0.47	0.04	0.01
σ=0.4	1.00	1.00	0.97	0.96	0.78	0.47	0.22	0.06	0.31	0.04	0.00
σ=0.5	1.00	1.00	0.97	0.96	0.48	0.47	0.14	0.00	0.00	0.04	0.01
σ=0.6	1.00	1.00	0.96	0.96	0.04	0.47	0.07	0.00	0.00	0.04	0.00
σ=0.7	1.00	0.99	0.96	0.96	0.00	0.47	0.00	0.00	0.00	0.04	0.00
σ=0.8	1.00	1.00	0.95	0.96	0.00	0.47	0.00	0.00	0.00	0.04	0.01
σ=0.9	1.00	1.00	0.95	0.95	0.00	0.47	0.00	0.00	0.00	0.04	0.00
mean	1.000	0.999	0.972	0.957	0.476	0.378	0.281	0.214	0.173	0.043	0.004

We next use eye-tracking time-series to evaluate the performance of saccade detection algorithms. In the publicly available dataset we use, eye movements have been manually annotated by two eye movement experts (Larsson et al., 2013). It is observed from the dataset that the two experts do not always agree with each other in the labeling of saccades, even when the data are of good quality. Here, we use the annotation by Nystrom to compare algorithms. The number of saccades in each record is indicated in the first column of Table 4. The results reported in Table 4 show that the proposed algorithm outperforms other algorithms. Nystrom's algorithm also performs well on this dataset. Overall, most algorithms perform well in detecting normal saccades when the data are of good quality. However, existing algorithms cannot reliably detect pathologically slow saccades correctly, which hinders the incorporation of saccade detection algorithms in clinical practice.

**Table 4.** Evaluation of saccade detection algorithms on eye-tracking time-series with saccades annotated by experts. F1 scores show the proposed algorithm outperforms other algorithms. The algorithm by Nystrom detect saccades similarly well.

id (#sacc.)	Proposed	Nystrom	Zemblys	Engbert	KF	König	VT	Friedman	DT	HMM	MST
1 (26)	1.00	0.98	0.98	0.93	0.98	1.00	0.94	1.00	0.83	0.96	0.04
2 (6)	0.91	0.80	0.80	0.75	0.80	0.80	0.71	0.80	0.67	0.73	0.00
3 (28)	0.90	0.88	0.84	0.83	0.52	0.82	0.65	0.88	0.65	0.37	0.18
4 (34)	0.93	0.87	0.93	0.75	0.84	0.89	0.77	0.91	0.77	0.73	0.07
5 (32)	0.93	0.95	0.95	0.86	0.89	0.93	0.94	0.81	0.87	0.85	0.03
6 (30)	0.98	1.00	0.98	0.94	0.97	0.97	0.98	0.97	0.87	0.89	0.22
7 (32)	0.97	0.97	0.94	0.95	0.94	0.95	0.95	0.95	0.87	0.90	0.00
8 (30)	0.95	1.00	0.92	0.90	0.85	0.91	0.93	0.89	0.86	0.83	0.11
9 (26)	0.89	0.89	0.92	0.92	0.96	0.82	0.75	0.30	0.85	1.00	0.17
10 (30)	0.98	0.97	0.88	0.76	0.86	0.67	0.87	0.83	0.82	0.76	0.26
11 (22)	0.95	0.95	0.64	0.82	0.71	0.50	0.89	0.90	0.90	0.64	0.02
12 (22)	0.91	0.95	0.72	0.91	0.55	0.59	0.42	0.84	0.71	0.46	0.09
13 (32)	0.98	0.98	0.94	0.89	0.96	0.92	0.94	0.94	0.79	0.90	0.02
14 (27)	0.94	0.90	0.86	0.90	0.92	0.88	0.65	0.30	0.68	0.92	0.46
mean	0.946	0.936	0.878	0.864	0.838	0.832	0.814	0.809	0.795	0.780	0.119

Note that the proposed method, and some other methods, perform better on the simulated data than on the real data. Some reasons for this may include measurement artifacts in the real data that make it more challenging, more complicated saccade waveforms, and the lack of absolute ground truth (owing to reliance on annotation for evaluation). Even if the parameters of the method are manually set, we do not expect the method to give a perfect F1 score for all datasets.

### Slow saccades

We show the proposed algorithm is also effective in detecting pathologically slow saccades. Slowing of saccades is indicative of lesions in specific areas of the brain and a powerful diagnostic tool in clinical use. However, existing saccade detection algorithms are not able to detect slow saccades properly. From five patients, we collected saccadic time-series with pathological slow saccades using the EyeLink 1000 Plus (SR Research Ltd). Four of the patients had supranuclear gaze palsy with symmetric bilateral saccade slowing; of these, three were diagnosed with PSP and one had spinocerebellar ataxia (genetically undiagnosed). The fifth patient had multiple sclerosis with an internuclear ophthalmoplegia (INO) causing slowed adducting saccades in only one eye.

**Figure 6.**
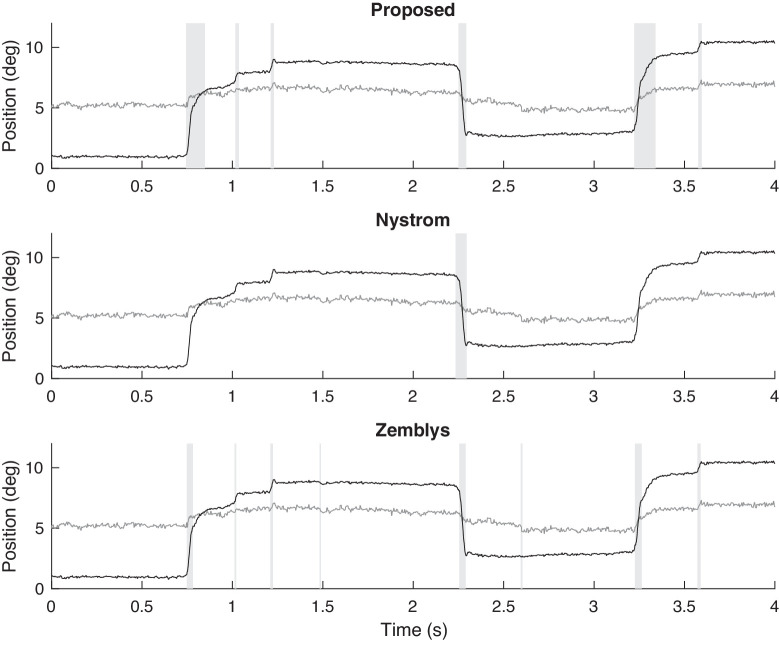
Example of saccade detection for a PSP patient who exhibits slow saccades, using the proposed algorithm and algorithms of Nystrom and Zemblys. Detected saccades are indicated by vertical bars shaded gray. Both the horizontal (black) and vertical (gray) components of the eye-tracking data are shown.

Figure 6 shows part of the eye-tracking time-series of one of the patients with PSP, which causes slow saccades. We compare the proposed algorithm with a state-of-the-art hand-crafted algorithm (Nystrom's) and machine-learning algorithm (Zemblys’). Detected saccades are highlighted in Figure 6 by vertical bars shaded gray. Nystrom's algorithm detects only one saccade and misses many slow saccades. Zemblys’ algorithm detects most saccades, but the end of each slow saccade is not correctly determined. To classify a saccade as slow, the peak velocity and the amplitude of the saccade must be be considered together. A slow saccade may be falsely classified as normal if the end of the saccade is not properly determined (i.e., the peak velocity might be correctly estimated, but the estimated saccade amplitude will be significantly underestimated). The result shows the proposed algorithm can detect slow saccades more accurately than these two algorithms.

**Figure 7.**
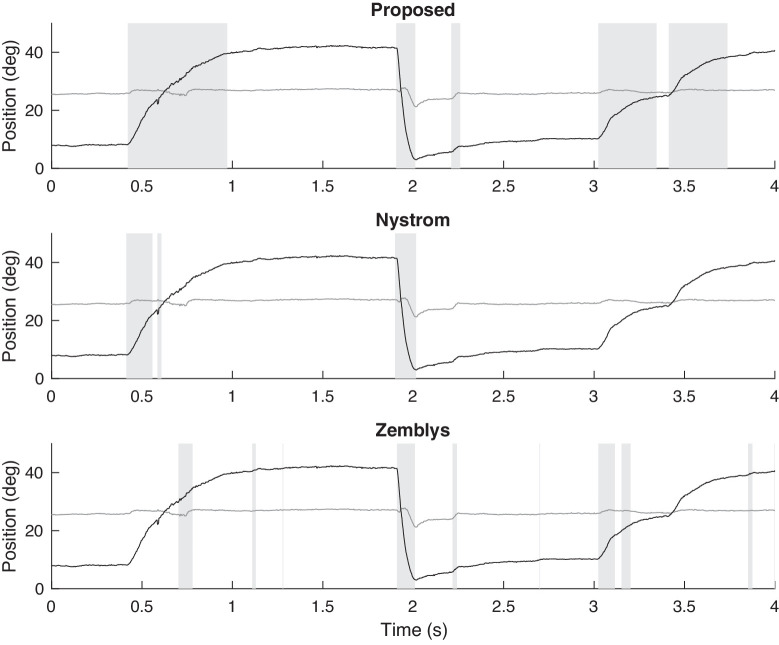
Example of saccade detection for an INO patient who exhibits slow adducting saccades and normal abducting saccades, using the proposed algorithm and algorithms of Nystrom and Zemblys. Detected saccades are indicated by vertical bars shaded gray. Both the horizontal (black) and vertical (gray) components are shown.

Figure 7 shows part of the eye-tracking time-series for the left eye of a patient with INO, who made a saccade to the right (adduction) and another saccade to the left (abduction). Patients with INO have injury in the medial longitudinal fasciculus. Consequently, the patient has slow adducting saccades and normal abducting saccades. This is an illustrative example because it shows that both Nystrom's and Zemblys’ algorithms can detect the normal abducting saccades properly. However, as shown in the previous example, they do not correctly detect the end of slow saccades. As a consequence, large slow saccades may be falsely classified as small normal saccades.

**Figure 8.**
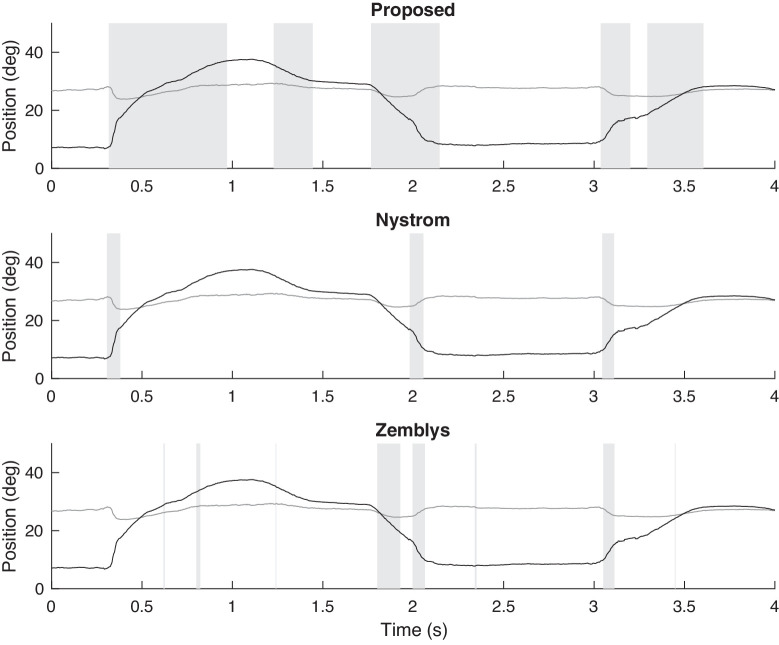
Example of saccade detection for a PSP patient who exhibits slow saccades using the proposed algorithm and algorithms of Nystrom and Zemblys. Detected saccades are indicated by vertical bars shaded gray. Both the horizontal (black) and vertical (gray) components are shown.

**Figure 9.**
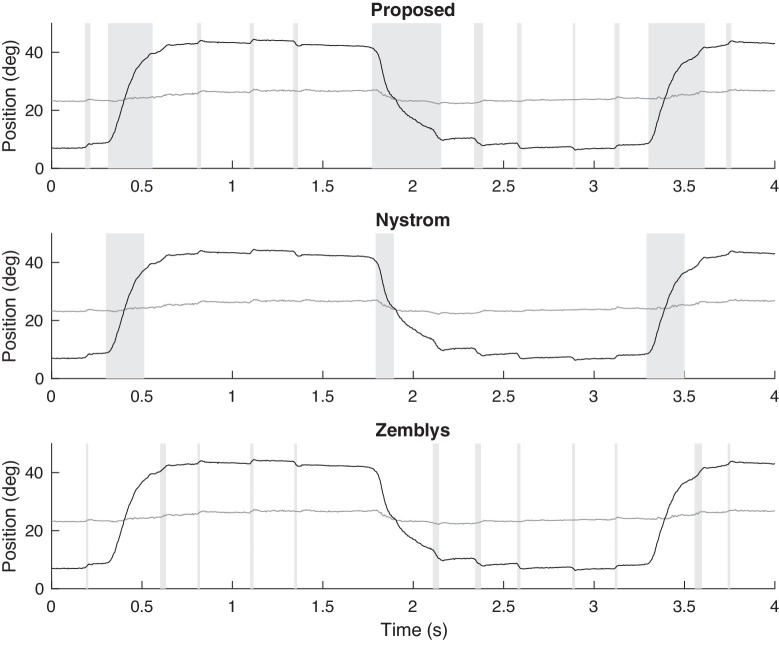
Example of saccade detection for a PSP patient who exhibits slow saccades using the proposed algorithm and algorithms of Nystrom and Zemblys. Detected saccades are indicated by vertical bars shaded gray. Both the horizontal (black) and vertical (gray) components are shown.

**Figure 10.**
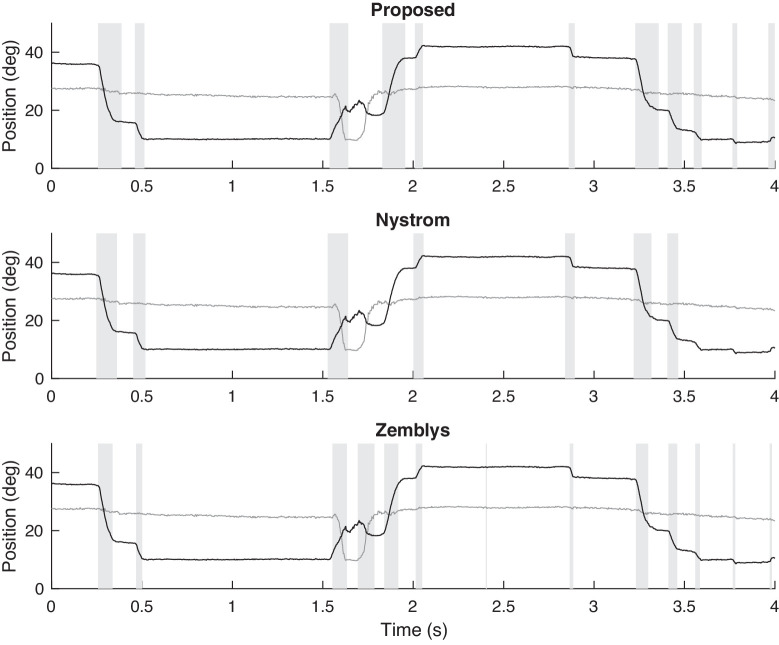
Example of saccade detection for a PSP patient who exhibits slow saccades using the proposed algorithm and algorithms of Nystrom and Zemblys. Detected saccades are indicated by vertical bars shaded gray. Both the horizontal (black) and vertical (gray) components are shown.

Eye-tracking time-series for the three additional subjects are shown in Figures 8–10 where similar results can be observed. The saccade main sequence data shown in Figure 1(b) is from the individual whose time-series data is shown in Figure 9. (To obtain the peak velocity and amplitude values shown in Figure 1(b), we used the proposed saccade detection algorithm as the first step.)

These five examples demonstrate that the proposed algorithm generally outperforms other algorithms for the detection of slow saccades. It is not surprising because parameters in Nystrom's algorithm are tuned for eye movements made by healthy subjects. The labeled training data used in Zemblys’ algorithm are also from eye movements made by healthy subjects. It is possible that changing parameters in Nystrom's algorithm or using labelled patients’ data in training Zembly's algorithm would detect slow saccades more accurately. However, Nystrom's algorithm has more than 10 parameters, and we usually do not have prior knowledge that a saccade is normal or slow, which complicates parameter setting and training in Zembly's algorithm. The proposed algorithm performs well on both physiologically normal and pathologically slow saccades without user input.

## Conclusion

In this article, we present an algorithm designed for the detection of both normal and slow saccades in realistically noisy time-series. Although there are algorithms that can detect normal saccades fairly well, most fail to accurately detect slow saccades. The proposed algorithm consists of two steps: nonlinear denoising and basic VT. For denoising of eye movement time-series, we define the objective (function 5) based on sparsity of the first-order and third-order temporal derivatives, and we develop an iterative optimization algorithm for its minimization. The velocity of the denoised time-series is mostly zero, except during saccades; thus, saccades can be easily detected via VT.

The denoising step is the most important part of the method. The use of the L1 norm and the combination of the first-order and third-order temporal derivatives in the formulation performs a type of nonlinear smoothing that reduces noise while preserving abrupt changes (“quasi-steps”) in a time-series, where the steps exhibit polynomial transition behavior.

We use both simulated saccades and real saccades generated by healthy subjects and patients to evaluate the performance of the proposed algorithm and ten other algorithms for saccade detection. For the detection of normal saccades, we demonstrate the proposed algorithm is as accurate, if not more accurate, than many other algorithms. We also show the proposed algorithm is capable of detecting slow saccades correctly; a process that is usually problematic and very valuable in clinical practice.

Note that in this work we consider the problem of saccade detection (including onsets and end-points) rather than the estimation of general saccade parameters such as direction, peak velocity, angle, and so on. We have studied the relative performance of various algorithms for saccade detection, but not for the estimation of general saccade parameters. For the estimation of such, it may be useful to reprocess the original data after saccades have been detected.

The proposed method is not intended for subjects with nystagmus or for eye-tracking data with smooth pursuit eye movements. Modifying the method to account for the presence of nystagmus and/or smooth pursuit eye movements remains as future work.
